# Magnetic-Based Human Tissue 3D Cell Culture: A Systematic Review

**DOI:** 10.3390/ijms232012681

**Published:** 2022-10-21

**Authors:** Inês Alexandra Marques, Carolina Fernandes, Nuno Tiago Tavares, Ana Salomé Pires, Ana Margarida Abrantes, Maria Filomena Botelho

**Affiliations:** 1 Coimbra Institute for Clinical and Biomedical Research (iCBR) Area of Environment, Genetics and Oncobiology (CIMAGO), Institute of Biophysics, Faculty of Medicine, University of Coimbra, Azinhaga de Santa Comba, 3000-548 Coimbra, Portugal; 2 Center for Innovative Biomedicine and Biotechnology (CIBB), University of Coimbra, Azinhaga de Santa Comba, 3000-548 Coimbra, Portugal; 3 Faculty of Pharmacy, University of Coimbra, Azinhaga de Santa Comba, 3000-548 Coimbra, Portugal; 4 Faculty of Science and Technology, University of Coimbra, Rua Sílvio Lima, 3030-790 Coimbra, Portugal; 5 Cancer Biology & Epigenetics Group, Research Center of IPO Porto (CI-IPOP), RISE@CI-IPOP (Health Research Network), Portuguese Oncology Institute of Porto (IPO-Porto), Porto Comprehensive Cancer Centre (P.CCC), Rua Dr. António Bernardino de Almeida, 4200-072 Porto, Portugal; 6 Clinical Academic Center of Coimbra (CACC), Azinhaga de Santa Comba, 3000-548 Coimbra, Portugal

**Keywords:** 3D cell culture, magnetic nanoparticles, spheroid, magnetic levitation, magnetic bioprinting, ring formation

## Abstract

Cell-based assays, conducted on monolayer (2D) cultured cells, are an unquestionably valuable tool for biomedical research. However, three-dimensional (3D) cell culture models have gained relevance over the last few years due to the advantages of better mimicking the microenvironment and tissue microarchitecture in vivo. Recent magnetic-based 3D (m3D) cell culture systems can be used for this purpose. These systems are based on exposing magnetized cells to magnetic fields by levitation, bioprinting, or ring formation to promote cell aggregation into 3D structures. However, the successful development of these structures is dependent on several methodological characteristics and can be applied to mimic different human tissues. Thus, a systematic review was performed using Medline (via Pubmed), Scopus, and Web of Science (until February 2022) databases to aggregate studies using m3D culture in which human tissues were mimicked. The search generated 3784 records, of which 25 met the inclusion criteria. The usability of these m3D systems for the development of homotypic or heterotypic spheroids with or without scaffolds was explored in these studies. We also explore methodological differences specifically related to the magnetic method. Generally, the development of m3D cultures has been increasing, with bioprinting and levitation systems being the most used to generate homotypic or heterotypic cultures, mainly to mimic the physiology of human tissues, but also to perform therapeutic screening. This systematic review showed that there are areas of research where the application of this method remains barely explored, such as cancer research.

## 1. Introduction

Pre-clinical cell-based assays have been a fundamental tool for biomedical, pharmaceutical, and biotechnology research, namely for the development of new diagnostic methods, drug discovery and screening, disease study, tissue engineering, and regenerative medicine, among others [1,2]. The use of cell-based assays allows us to minimize the extensive, expensive, and ethical-related issues associated with the use of animal models for research purposes [2]. So far, most of these assays are still widely based on bidimensional (2D) cell culture models, considering the low cost, simplicity, and reproducibility [3,4]. The standard process for drug development starts by testing the drug in a 2D cell culture model followed by animal testing and lastly, the clinical trials phase. However, about 90% of new drugs fail in clinical trials, mostly due to the ineffectiveness of existing preclinical tests in representing the complex and natural human microenvironment and, consequently, in predicting the human biological response to molecules [5].

### 1.1. Three-Dimensional (3D) Cell Culture Systems

Three-dimensional (3D) cell culture models are an emerging area with several benefits and advantages to overcome the aforementioned 2D cell culture limitations. Generally, 3D cultures allow for conducting cancer research and drug screening in a microarchitecture that is more similar to human tissue. The genetic analysis of samples allowed us to establish a greater correlation between the profiles of human tissues and 3D cell cultures, compared to animal models [4]. Thus, 3D cell cultures present several advantages: (i) a microenvironment and microarchitecture similar to in vivo, presenting more biological and physiological relevancy; (ii) a complex structure with several cell–cell and cell–extracellular matrix (ECM) interactions, closely mimicking the intracellular communications; and (iii) the possibility to have different access to nutrients and oxygen as occurring in human tissues. All these attributes make the 3D systems a good simulator both to move forward in translational preclinical research, as well as to allow reduction in the use of animal models [6,7].

The term 3D culture has been widely used in the bibliography to describe several types of 3D structures. Diverse sources can be used for 3D cultures such as cell lines, stem cells, primary tissues, and embryonic whole organs, among others. Based on cellular origin, aggregate morphology, and culture methods, Weiswald and colleagues proposed a classification of 3D structures into four distinct classes: multicellular tumor spheroids, tumorspheres, tissue-derived tumorspheres, and organotypic multicellular spheroids. However, the same terminology is still widely used for different groups, such as the use of the term spheroid to refer either to multicellular spheroids or to tumorspheres [8]. Spheroids are derived from primary sources or cell lines and do not have the ability to differentiate or self-organize, allowing for an easy distinction between them and the organoids. Therefore, spheroids have the advantage of allowing the removal or addition of cell types and elements relevant to the type of investigation to be carried out. Organotypic multicellular spheroids, often called organoids, are derived from biopsies or tissues and have the capability to self-organize into differentiated and complex structures, capable of recapitulating some physiological functions similar to the source. Considering the diversity of cell types that constitute the spheroids, they can be classified as homotypic or heterotypic, also known as co-cultures, if they are composed of only one or more than one cell type, respectively [6,9].

Several 3D cell models have been proposed, such as the hanging drop technique, the use of scaffolds, hydrogels or cell sheets, microfluid systems, rotatory and bioreactor systems, and, more recently, the magnetic-based 3D (m3D) cell culture [2,6,10,11,12].

### 1.2. Magnetic-Based 3D (m3D) Cell Culture Technology

This magnetic-based technology consists of incubating cells in 2D apparatus with magnetic nanoparticles (MNP), named NanoShuttle^TM^-PL, composed of a mixture of iron oxide, gold, and poly-L-lysine nanoparticles. These MNP will bind electrostatically and non-specifically to the cell membrane to magnetize them. Then, magnetized cells are enzymatically dissociated and transferred to a 3D apparatus, according to the methodology described for each m3D method. Then, cells will be exposed to magnetic fields generated by neodymium magnets to induce them to aggregate into 3D structures [1].

**Figure 1:**
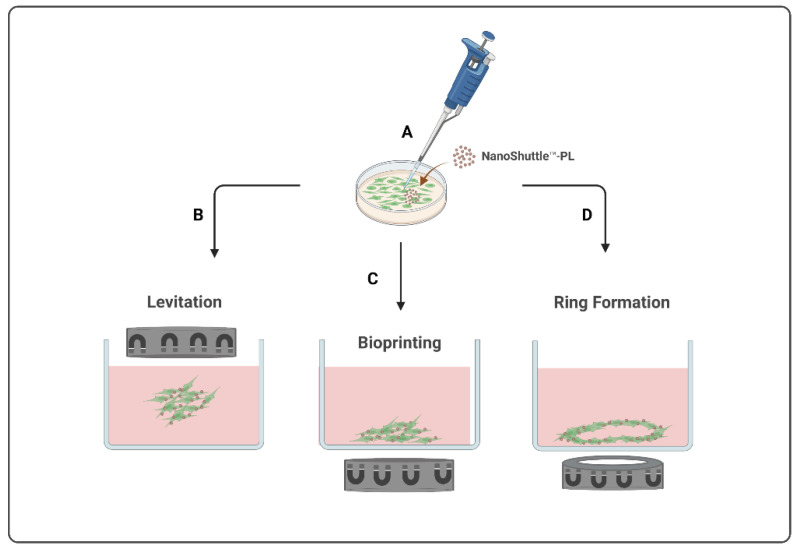
Schematic illustration of magnetic-based 3D (m3D) systems to develop 3D structures. This method starts with the magnetization of the cells (**A**) to generate 3D structures by levitation (**B**), bioprinting (**C**), or by ring formation (**D**).

Currently, there are three types of magnetic-based models available, presented in Figure 1, all starting from the magnetization of the cells (Figure 1A) to create different final structures by levitation, bioprinting, or ring formation. The m3D levitation method (Figure 1B) consists of seeding the cells and then placing the magnets atop the plates to promote the levitation of magnetized cells in a liquid–air interface where they will aggregate into 3D structures by cell–cell and cell–extracellular matrix interactions [1,13]. The m3D bioprinting method (Figure 1C) consists of placing the magnets under the plates, where magnetized cells were seeded, to promote cell aggregation and matrix formation by printing 3D structures at the bottom of each well. The most recent m3D method, ring formation (Figure 1D), consists of firstly levitating the magnetized cells to allow for the formation of aggregates with ECM, followed by a second step to disintegrate these 3D cultures into dispersed cells, and, lastly, placing these plates atop a magnetic unit with ring-shaped neodymium magnets to induce cell aggregation in a toroidal shape [1]. These magnetic-based techniques are an easy procedure to implement and standardize using diverse cell types, allowing for a fast and consistent spheroid formation as well as a controlled cellular movement and aggregation [1,13,14] Moreover, since spheroids are able to produce their own endogenous ECM during their formation and aggregation process, there is no need to use an artificial matrix [1,13].

These advantages have driven the development of studies using m3D-based cultures over the years for a variety of research purposes [15]. Therefore, this systematic review aims to bring together studies focused on m3D-based cell cultures to explore the different 3D models already developed with this technology, including tumor models, as well as the characteristics of the 3D structures formed, the most suitable materials to use, the implemented analysis techniques, and the limitations and possible applicability of these methods to create heterotypic 3D cultures. The integration of all this knowledge may be of interest to the progress of human health research.

## 2. Materials and Methods

This systematic review was carried out according to guidelines proposed by the Preferred Reported Items for Systematic Review and Meta-Analysis (PRISMA) statement [16].

### 2.1. Literature Search Strategy

We sought to identify studies using m3D cultures developed by levitation, bioprinting, or ring formation. A systematic and comprehensive search was conducted in three main electronic databases—Medline (via PubMed), Web of Science, and Scopus—using the search queries presented in Table 1. The filter of the English language was applied. The search was done on 3 February 2022.

### 2.2. Study Selection

The selection of studies was based on several eligibility criteria. The articles should meet the following criteria to be included: (i) use the m3D cell culture method; (ii) have in vitro 3D cell cultures of spheroids or organoids or protocols for their establishment; (iii) use the NanoShuttle^TM^-PL or previous commercial name; and (iv) be an original article written in the English language. The articles falling under the following criteria were excluded: (i) other non-experimental articles; (ii) studies non-related to the topic; (iii) articles using other nanoparticles; (iv) in vivo studies; (v) not written in English; (vi) full article not available.

### 2.3. Data Extraction and Analysis

**Figure 2:**
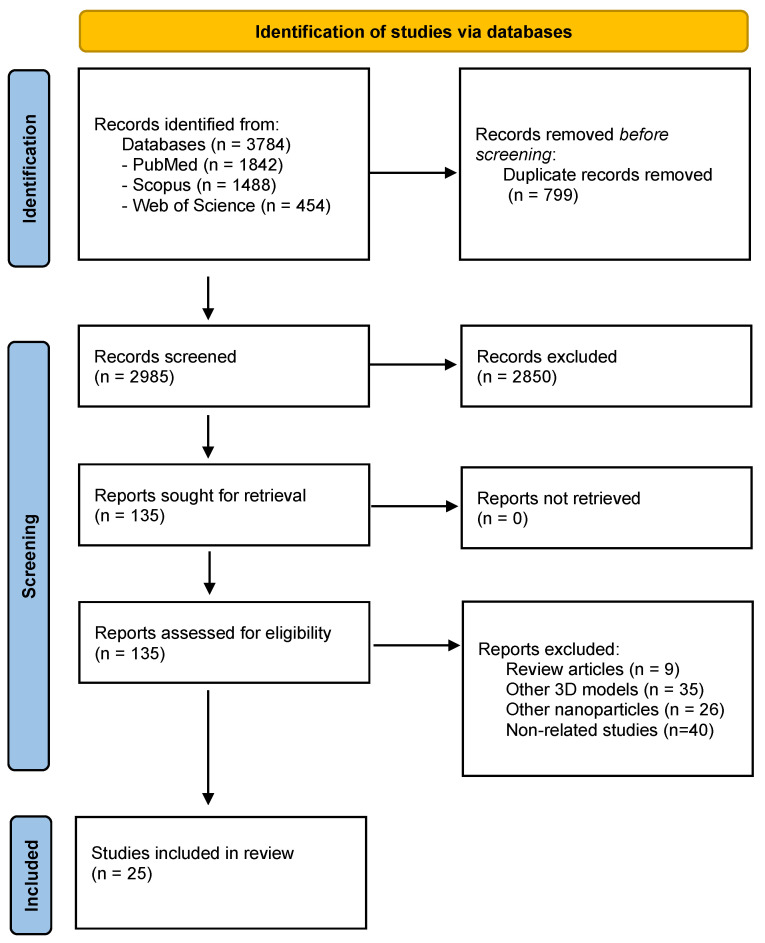
Prisma Flow diagram of the methodology used for literature search and studies selection in this systematic review.

All the articles identified from databases were first downloaded into a public reference manager software (Mendeley Desktop v1.19.4) and duplicates were eliminated. Then, two reviewers independently screened titles and abstracts for identifying potentially relevant studies, based on eligibility criteria. The selected articles were further read in full to ensure eligibility. In the case of disagreements between the two reviewers, a third reviewer was consulted. The PRISMA flow chart for the selection process of studies included in this review is explained in Figure 2.

Qualitative data were collected from each eligible reference, in particular, data regarding: (i) authors and publication year; (ii) m3D model used; (iv) homotypic or heterotypic 3D model; (v) 3D mimicking tissue or disease; (vi) cell lines; (vii) use of scaffolds; (viii) applications; and (ix) observations regarding the model.

## 3. Results and Discussion

As aforementioned, the guidelines presented in PRISMA statement were followed to conduct this systemic review. Based on that, as shown in Figure 2, we identified 3784 potentially relevant articles from the databases, of which 799 duplicates were eliminated. After title and abstract screening, 2850 studies were withdrawn. Then, 135 studies were read in full, and the following were excluded: 9 review papers, 35 papers presenting other 3D models, 26 papers using other nanoparticles, and 40 studies not related to the magnetic technology of interest. In the end, a total of 25 studies were selected. Data extracted from these studies are gathered and summarized in Table A1. The studies collection clearly showed that the use of this methodology is recent since the first publication refers to 2010. The number of publications has been increasing over the last few years.

The studies were grouped and discussed according to the main topics: (1) m3D culture method; (2) composition of m3D culture; (3) biocompatibility of MNP; (4) use of scaffolds or not; (5) formation period of 3D structure and longevity in culture; (6) tools and methodologies applicable to m3D cultures; (7) size of the m3D structures and core features; (8) cells behavior and intercommunication in m3D cultures.

### 3.1. m3D Culture Method: Levitation, Bioprinting or Ring Formation

The use of 3D cell cultures has been growing and updated over recent years. Despite the improvement of techniques for creating 3D structures, the size, shape, and morphology of 3D structures are challenging to control. In addition, the risk of disintegration of these structures is presented as one of the biggest limitations, occurring even with the use of ultra-low attachment (ULA), and, especially, when multiple manipulations are required during experiments. The m3D culture technology, only commercialized by Greiner Bio-One GmbH, was designed to address these problems through the development of three main models, namely levitation, bioprinting, and ring formation. Magnetic-based models allow us to effectively produce homogeneous 3D aggregates and with desirable compactness [1,17]. Of the twenty-five studies included in this review, nine studies used only magnetic levitation [17,18,19,20,21,22,23,24,25], eleven studies used only the bioprinting method [14,26,27,28,29,30,31,32,33,34,35], and three studies used both methods [15,36,37]. Only two studies used the magnetic ring formation method [22,38]. Therefore, the magnetic bioprinting method is the most often applied, followed by the magnetic levitation method and, lastly, by the magnetic ring structure formation. The results regarding the ring formation method are not surprising considering that it has only been available most recently. Regardless of the methods used, it was generally demonstrated that all these models provided excellent reproducibility, allowed easier handling of spheroids, and allowed for a wide range of experiments to test various physical or chemical agents [26,30].

As aforementioned, the MNP used in these m3D methods to promote the electrostatic attaching to the cell membrane is the NanoShuttle^TM^-PL, consisting of gold and iron oxide nanoparticles cross-linked with poly-L-Lysine and with a diameter of less than 50 nm. These MNP magnetize the cells by electrostatically attaching to cell membranes within a short incubation time [21]. In these m3D systems, the use of the Nanoshuttle^TM^-PL is complemented by a specific magnetic field depending on the purpose. For magnetic levitation, a magnetic drive is available to be placed atop the magnetized cells; whereas, for magnetic bioprinting, there is a concentration drive to put above the magnetized cells. It is also available as a drive with ring-shaped magnets that can be complemented with the levitation or bioprinting driven to generate ring-shaped 3D structures. There are different kits commercially available, with magnetic drives and plates varying according to the intended method. The 24-well plates were the most used for studies using the levitation method; whereas, the 96-well plates were the most used for the bioprinting method. More precisely, nine studies used the 24-well Bio-Assembler Kit [15,23,24,25,26,30,32,37,39], eight studies used the 96-well Bioprinting Kit [14,27,28,29,31,33,35,36], two studies used both 6-well and 24-well Bio-Assembler Kit [17,19], one study used only the 6-well Bio-Assembler Kit [34], and one study used the 96-well Ring Drive for ring structure formation [38]. There is an additional study that used tissue culture Petri dishes covered by a top cover with an attached neodymium magnet [22]. Daquinag and his team observed that the use of different plate sizes could influence the establishment of 3D culture formation with more uniform and solid m3D spheroids formed when smaller cell culture plates were used [19]. Apart from the plate size, it was generally pointed out that the use of flat-bottom ultra-low attachment plates was associated with a maximum levitation efficiency [13].

### 3.2. Composition of m3D Cultures: Homotypic or Heterotypic

Generally, eleven studies developed homotypic cultures and fourteen studies were focused on generating heterotypic cultures. As aforementioned, homotypic cultures are made of only one cellular type, whereas heterotypic cultures are generated by more than one type of cells [6].

In general, it is possible to generate homotypic and heterotypic cultures using the magnetic levitation model. The sources and types of cells used among all studies included are very heterogeneous, showing that this model can be applied to several types of m3D aggregates. Eleven studies were dedicated to the generation of homotypic m3D cultures, with the cell types used depending on the research objectives. Human dental pulp stem cells (hDPSCs) were used to produce the bioprinting method innervated secretory epithelial organoids to assess epithelial regenerative potential after transplantation in ex vivo models [27]. Spheroids of hDPDCs were also developed by magnetic levitation for evaluating the therapeutic efficiency in using these cells for regenerative medicine [17]. Human vascular (aortic) smooth muscle cells (VSMCs, PH35405A cell line) were used to develop a better representation of the in vivo environment of VSMCs [18]. Human embryonic kidney cells (HEK293 cell line) and human primary tracheal smooth muscle cells (SMC) were used for developing m3D homotypic ring-shaped structures for conducting drug toxicity screening [38]. Human fetal osteoblast cells (hFOB 1.19) were used for bioprinting m3D homotypic spheroids to investigate normal bone physiology and bone tissue engineering and regeneration [14]. Glioblastoma cells (U87) were used for bioprinting m3D spheroids to mimic the wound healing process, with the purpose of testing new wound dressings containing the *Plantago australis* hydroethanolic extract [33]. In addition, m3D homotypic cultures of human telomerase reverse transcriptase (hTert)-immortalized retinal pigment epithelium (RPE) cells, squamous cell carcinoma lines (HPV negative or positive), and osteosarcoma cells (FUCCI cell line) were produced to study the hypoxia-dependent radioprotective phenotype; however, RPE cells failed in forming spheroids [32]. Primary normal human fibroblasts (NHF) and skin squamous cell carcinoma cells (SCC13) bioprinted into m3D spheroids mimicked the complex design of the 3D architecture and ECM of the human skin [34]. There were also studies using animal cell lines to develop m3D homotypic models for drug screening and to study the biological response: (i) pancreatic ductal adenocarcinoma (PDAC) spheroids made of PDAC DT66066 cells were used to evaluate the effects of inertial cavitation, in the presence or absence of chemotherapy; (ii) m3D of cow trophectoderm-1 (CT-1) cells were developed to test if the MNP could be used to improve cell attachment and proliferation; and (iii) m3D of C2C12 mouse myoblasts to investigate the organization and composition of the ECM within 3D tissue models [21,26,29]. Hence, most of the homotypic m3D cultures were developed to access the normal physiological response, interaction with ECM, and the organization of in vivo tissues and only few were developed for conducting experiments for anti-cancer drug screening.

The development of both homotypic and heterotypic m3D cultures, within the same study, were observed in most of the articles included in this review. If we focus on heterotypic m3D cultures, there are fourteen out of the twenty-five studies that describe the generation and use of heterotypic cultures. Typically, heterotypic cultures are composed of one or more tissue-like cells. The use of stromal cells from connective tissue cells of the organ or tissue to be mimicked is often used to promote natural cell aggregation and support tissue function when in spheroid or organoid structure. Examples of these commonly used stromal cells are epithelial and mesenchymal cells, and fibroblasts, among others. The studies included a huge variety of methodologies to attain heterotypic m3D cultures. Two main approaches are often used: (i) different types of magnetized cells are seeded together at the beginning of the 3D culture and forced to aggregate into spheroids; or alternatively, (ii) each cell type is firstly grown into homotypic spheroids and then each homotypic spheroid is sequentially harvested and assembled to form the 3D heterotypic spheroids [23,24].

As previously stated, the heterotypic spheroids are defined as a 3D structure consisting of several cell types to allow for a better mimic of in vivo physiological conditions. Thus, the cell types and cell sources used are quite diverse, as expected considering the wide range of applications done by several authors. Spinal cord cells from Long Evans rat embryos, more specifically neurons, oligodendrocytes, astrocytes, neural precursor cells, and microglia were used to develop a 3D microphysiological model of the central nervous system [28]. The use of mouse preadipocyte cells and murine endothelial cells allowed for the formation of adipospheres as a model of white adipose tissue development and growth [19]. Heterotypic m3D cultures constituted by human ovarian cancer cells (CAISMOV24 cell line) and peripheral blood mononuclear cells (PBMCs), such as monocytes, lymphocytes, and macrophages, were used to study the growth of a low-grade serous ovarian carcinoma and the role of the interactions with immune cells in the tumor microenvironment [15]. Pancreatic tumor cells isolated from KPC-transgenic mice and murine embryonic fibroblasts were combined in m3D cultures to study ultrasound therapy [30]. Moreover, there are two published protocols to describe how to build m3D cultures that are adapted for several types of cells, either from human or animal sources. One of the protocols presented the general procedures and summarizes the types of cells already grown into m3D cultures, showing the differences in shape and size depending on the cell line used as well as initial cell density [13]. The protocol published by Leonard and Godin [36] presented the steps to develop 3D spheroids using the magnetic and bioprinting method to mimic the features of in vivo lesions of breast cancer, including the role of the immune system.

The m3D was also used to develop organoids, also named organotypic multicellular spheres. Organoids of human hematopoietic stem cells were generated by joining human bone-marrow mesenchymal stem cells (MSCs), non-tumoral dermal microvascular endothelial cells (CC-2811 cell line), and umbilical cord blood-hematopoietic stem cells (HSCs) to mimic the in vivo microenvironment and evaluate their potential in regenerative medicine [39]. Human astrocytes and glioblastoma cells were combined to form m3D cultures to access the in vivo protein expression [22]. Human epithelial cells (EpiCs), smooth muscle cells (SMCs), pulmonary fibroblasts (PFs), and pulmonary endothelial cells (PECs) were grown together to develop an m3D model of the bronchioles that mimic the native extracellular matrix [23]. Porcine valvular interstitial cells and endothelial cells were combined to generate an aortic valve m3D heterotypic cultures for the study of heart valve biology [24]. There is also a protocol for the development of m3D heterotypic cultures (adipospheres) of white adipose tissue (WAT) by joining murine embryonic preadipocytes, endothelial cells, and cells from the stromal vascular fraction of original tissue, allowing for the study of the native structure and function and their use for high-throughput studies of WAT [25]. M3D cultures of human pancreatic β cells (endoC-βH3 cell line) and VEGF pre-screened umbilical vein endothelial cells (HUVEC cell line) allowed for the investigation of the interaction between both cell types [31]. Tuberculosis granulomas were resembled into m3D cultures using human alveolar macrophages and autologous CD^3+^ T cells to study the host/pathogen pathways and the immune response involved in the infection process [37]. Chicken hepatocytes and non-parenchymal cells were used to establish a proper hepatic inflammatory model for testing potential proinflammatory molecules [35].

Generally, most of the heterotypic cultures mentioned in the included studies are comprised of, at least, one tissue cell type (tumoral or non-tumoral) and the stromal cells aiming for a better representation of the in vivo microenvironments, inclusive of the production of ECM. The main goal in establishing the m3D heterotypic cultures is the representation of several tumors’ microenvironments and physiological functions. Only a few models were developed for drug screening and cancer research, probably due to the high complexity of mimicking in vitro the carcinogenesis process and all the players.

### 3.3. Biocompatibility of Magnetic Nanoparticles (MNP)

The m3D method is based on the use of MNP to magnetize the cells followed by exposure to a magnetic field to force them to aggregate. The MNP is commonly used at a recommended concentration of 8 µL/cm^2^ of culture area for all the m3D systems [13,36]. However, a slight difference in the concentration used among the few studies was noted, with a higher MNP concentration added to cells for the magnetic levitation method than for the bioprinting method.

Moreover, the MNP should comprise the following characteristics: be non-toxic, not affect cell proliferation or metabolism, and not induce pro-inflammatory responses or oxidative stress [1]. The Nanoshuttle™-PL biocompatibility was tested in almost all the reviewed studies to ensure that it did not interfere with the experiment under development. Generally, the non-cytotoxicity of the MNP was reported, since MNP were not toxic to the cells and did not interfere with the substances tested in the m3D structures [14,27]. Apart from the electrostatic binding to cell membranes, it was reported that MNP is incorporated into the cell cytoplasm, more precisely inside of endocytic vacuoles. No MNP was found in the cells’ nucleus. The results obtained by transmission electron microscopy of cells incubated with MNP for 12 h showed that the MNP incorporation did not induce cell architectural abnormalities [26]. Cell morphology, proliferation, or viability were not affected by the MNP, with more than 90% of viable cells after 3 days of culture [27]. Moreover, the MNP did not induce intracellular oxidative stress or any inflammatory responses [26]. Thus, the overall cytotoxicity of these MNP can be considered negligible, since the cell metabolism, measured by the levels of pyruvate and lactate, was not affected by the presence of MNP during incubation or when exposed to a magnetic field [29].

Concerned with the elimination process, in general, MNP were released from spheroids to the medium in a fast and steady way during long-term cultures [27]. Abou and colleagues verified that the release of MNP occurs after eight days of m3D cultures growth [26]. An improvement in the attachment of cell clumps when m3D systems were applied has been proved, pointing out the potential of incorporating this methodology into experiments involving cells that are difficult to grow [29]. No significant variations were verified in DNA fragmentation, a cell survival indicator, within m3D structures, namely in pseudo-islets [23,24]. Thus, the overall results pointed out by several authors confirm the MNP biocompatibility, as well as its beneficial role in promoting a quick and effective cell attachment when using cells difficult to grow into a 3D shape.

### 3.4. Use of Scaffolds in m3d Cell Cultures

The 3D cell culture models can be classified in scaffold-based or scaffold-free, if a scaffold is used or not, respectively, to establish the m3D structure. The magnetic-based model is also compatible with the two approaches, with 6 out of 25 studies using scaffold-based methods. Generally, the combination of a magnetic model with a scaffold was beneficial for conducting experiments using levitation and bioprinting methods. Concerning magnetic levitation, they were particularly used for 3D homotypic cultures development [18,22]. On the other hand, the use of scaffolds was also applied in the bioprinting method to develop both homotypic and heterotypic 3D cultures [28].

The scaffolds used for the experiments were quite different, including in their origin. Two scaffolds used in the experiments were of synthetic origin. One is thermoresponsive and made of Poly(urethane acrylate)-poly(glycidyl methacrylate to provide regulation of the cellular alignment and cell sheet transfer [21]. The other is made of polyvinyl alcohol (PVA) solutions, combined with semi-solid *Plantago australis* hydroethanolic extract (PAHE), to produce wound dressings and provide a continuous drug release source [33]. A m3D culture has also been produced by magnetic levitation in the presence of a natural hydrogel composed of M13-derived bacteriophage particles, displaying a ligand peptide (termed RGD-4C) that targets αv integrins, magnetic iron oxide (Fe_3_O_4_, magnetite), and gold nanoparticles [22]. Bowser and Moore [28] bioprinted m3D cultures in combination with a synthetic three-dimensional hydrogel construct, made from naturally-sourced poly(ethylene glycol) diacrylate and Matrigel, which provides external guidance that directs neurite projections. Thus, it seems that the MNP and the m3D systems are compatible to be complemented with a huge range of scaffolds without compromising the efficiency of the magnetic fields.

### 3.5. Formation Period of m3D Structure and Longevity in Culture

Typically, magnetized cells aggregate into 3D structures within the first 15 min of exposure to the magnetic field and complete the aggregation within 24 h [25]. The precise incubation time needed for this method varies according to the magnetic model used, the type of 3D structure intended, the cell lines used, and the specific characteristics of each experiment, such as the use of a scaffold. No substantial variations were observed between the homotypic and heterotypic 3D cultures. Adine and colleagues reported that cells aggregated in homotypic 3D bioprinted cultures during the first 15 min of culture exposed to a magnetic field [27]. Additionally, levitated m3D homotypic and heterotypic cultures had an immediate aggregation of cells by the moment they were exposed to an external magnetic field [13]. Round-shaped structures developed with both levitation and bioprinting methods started to aggregate during the first 3 h under a magnetic field and were fully formed after 24 h [14,15]. Thus, several studies have shown that m3D structures formed faster comparatively to non-magnetic 3D cultures, which usually take between 24 to 48 h to form homogeneous and compact 3D structures. Moreover, the m3D structures are more consistent [17,27].

The longevity of the 3D cultures varies among experiments but, in general, the 3D structures obtained by magnetic methods presented a more consistent morphology and cell aggregation during 7 days [13,17,27]. Effectively, Adine and colleagues observed that 3D structures produced at the same time, by other non-magnetic methods, started to disintegrate after around 3 days of culture [27]. Considering these features, most of the studies chose to maintain the m3D cultures for 7 days, independently of the magnetic method applied, replacing the cell culture medium during this period. The 3D structures showed a typical growth progression over days, similar for all cell lines [13,17]. Daquinag and colleagues showed that it is also possible to maintain m3D cultures for up to 45 days, under a magnetic field, with most of the cells remaining viable [19]. The maximum longevity for m3D cultures was 12 weeks, with the integrity of the 3D structures remaining intact even after removing the magnetic field. These m3D cultures also exhibited extracellular matrix formation, no variations in phenotype, and maintenance of cell viability in the whole structure, which denotes that this methodology is suitable for long-term multicellular experiments [22]. Thus, the m3D system seems to be a good tool to evaluate long-term effects and conduct repetitive experiments, constituting an effective alternative to animal studies.

### 3.6. Tools and Methodologies Used for the Analysis of m3D Cultures

The challenging analysis of 3D cell cultures often relies on using microscopy techniques for image acquisition and evaluation measurements. These tools are mostly applicable for: (i) analyzing cell proliferation, viability, and metabolism; (ii) evaluating the morphology, size, volume, sphericity, and aggregation of the m3D structures; (iii) quantifying the global DNA content and the expression levels of mRNA; (iv) analyzing the intercellular communications and the formation of ECM; and (v) assessing the maintenance of phenotype and function, among others. Almost all studies included histological analysis by immunohistochemistry (IHC) and fluorescence, confocal, and electron microscopy, followed by image analysis in ImageJ software [13,15,17,19,23,24,26,27,29,30,31,32,37,39]. These techniques can be considered the standard techniques for analyzing all types of m3D cultures, since the MNP does not interfere with immunofluorescent assays, either increasing signal noise or interfering with fluorophore detection [13]. However, sometimes the presence of the MNP within the cells can interfere with the colour of the markers used in the IHC analysis, due to the MNP’s brown color. Moreover, the m3D structures are usually dense, which can make the microscopic analysis difficult, and inclusive of confocal images. The combination of both techniques, namely confocal imaging and IHC analysis, can help overcome these issues. Besides these techniques, PCR/qRT-PCR are also used for analyzing gene expression [14,17,24,27,39].

### 3.7. Size of the m3D Structures and Core Features

Magnetic-based 3D cell cultures vary in size. In general, magnetic bioprinting and levitation methods allowed us to obtain spheroids with a diameter between 300 µm and 1 mm. Otherwise, the ring structures formed were bigger and macroscopic, reaching a diameter of 4 mm, which facilitates its measurements and allows for the use of this method to produce larger 3D structures [38].

For instance, considering homotypic models, bioprinted spheroids of pancreatic ductal adenocarcinoma cells had a median diameter of 830.80 µm and an average thickness of 300 to 350 µm [26]. Bioprinted organoids of human dental pulp stem cells had a diameter of around 1 mm [27]. Otherwise, bioprinted osteoblast spheroids had only an average size of approximately 100 and 350 µm after 3 and 14 days of culture, respectively [14].

If we consider the heterotypic 3D models, the bioprinted spheroids of pancreatic ductal adenocarcinoma achieved a mean area of only 300 µm after 3 days of culture [30], and the levitated cultures of the aortic valve were approximately 500 µm thick [24]. These variations are mostly associated with the cell types used, but also depend on other factors, such as the starting cell number, culture medium volume, MNP concentration, and culture period, among other specific-inherent factors of each experiment. In fact, Haisher and his team showed that the size of the m3D spheroids developed with A549 cell line was dependent on the starting cell number and culture medium volume, with spheroids size increasing with the increase in starting cell number [13]. Apart from size, cell seeding density also influenced the shape and formation period of the m3D cultures [17]. Therefore, it is mandatory to optimize these features for each type of m3D culture established [13]. Furthermore, in the case of heterotypic m3D cultures, it is necessary to optimize not only the cell concentration, but also the proportion of each cell type. The analysis of the morphology of the m3D structure formed and the global DNA content could also allow for defining the best cell density for each cell type, as well as the optimal concentration of MNP needed [39].

**Figure 3:**
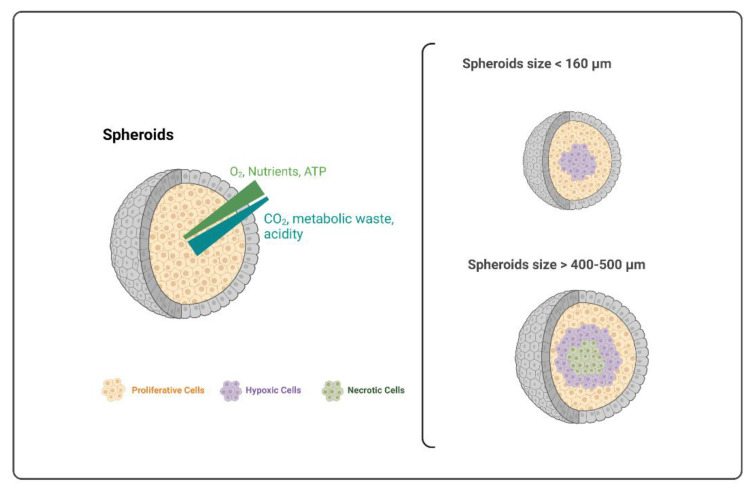
Representation of spheroid constitution and the core features according to their size.

The size and shape of 3D cell cultures are important factors to consider because they influence core features, such as hypoxic zones and necrotic cores. It has been stated that spheroids with a diameter larger than 160 µm start to develop a hypoxic nucleus and, if they reach sizes between 400 and 500 µm, their nuclei become necrotic. In fact, an increased spheroid size leads to lower oxygen flow and nutrient access, with the consequent appearance of hypoxic and necrotic zones in the core. This is illustrated in Figure 3. Unfortunately, the definition of the maximum size at which 3D structures developed hypoxic or necrotic core cannot be strictly defined, since they were also dependent on the cell type as well as the platform used for generating the 3D aggregates [1,14,40].

In m3D models, the hypoxic center was observed for homotypic spheroids of several cancer cell lines presenting a diameter higher than 500 µm, with the development of hypoxia at a distance of 100–110 µm from the periphery of the spheroid. In these cases, cell proliferation was limited to the outer 100 µm [32]. Moreover, Tseng and colleagues [25] do not recommend the levitation of spheroids with a diameter superior to 5000 µm because cell death and necrosis begin to occur in spheroids’ core. Moreover, cell death was also observed in the center of magnetically levitated spheroids formed with a starting cell density higher than 2.5 × 10^5^ cells [19]. The presence of a necrotic zone and the lack of spheroid vasculature are convenient when mimicking a first-stage tumor microenvironment since they start to grow vascularly till stabilizing their size [1]. For example, PDAC m3D spheroids developed by Leenhardt et al. illustrated the similarities between spheroids and a human PDAC tumor, such as the presence of the necrotic core, the increased cell viability from the center to the periphery of spheroids, and the complex interaction between cancer cells and active fibroblasts to produce collagen, resulting in the basis of the tumor microenvironment [30]. However, when it comes to non-tumor models, this necrotic zone should be avoided, since it does not represent the normal physiology of the human-like tissue. Thereby, it is recommended to avoid very large spheroids when working with non-tumor cells, which can be achieved by changing the seeding cell density to produce smaller spheroids with small necrotic zones [1,14,40]. Almost no non-tumor structure formed presented a necrotic core. This is a crucial observation for non-tumor models because it also demonstrates the maintenance of cell viability and the non-toxicity of the MNP used to generate the 3D structures [39].

### 3.8. Cell Behavior and Intercommunication in m3D Cultures

In a general way, the selected studies produced robust, viable, and consistent 3D structures, able to synthesize their own ECM, which is responsible for cell adhesion, cell-cell communication, and differentiation. Therefore, the application of a magnetic model to form 3D cell cultures is suitable for supporting cell–cell and cell–ECM interactions and, most importantly, for allowing cell proliferation and differentiation. The cell behavior and intercommunication determine several characteristics of the spheroids, such as their shape and morphology. The m3D structures obtained by Souza-Araújo et al. [15] evolved irregularly and generated regions with variable amounts of aggregated cells and spindle-like elongated shapes. In the end, the morphological analysis defined the final arrangement of the cells as papillary and revealed the presence of glandular-like structures. The changes in morphology show that the use of MNP and the magnetic field to which the magnetized cells are exposed are not the only factors that determine the final shape of 3D cell aggregates. The intrinsic capacity of the growing cells to organize themselves and determine their final arrangement plays an important role, allowing cell lines to reveal certain histological differentiation. Thus, the shape and morphology of m3D structures can vary depending on the cell lines used. For example, the morphology of bioprinted 3D osteoblast spheroids was regular and showed a stable cellular aggregation, allowing for the formation of compact surface spheroids with close cell–cell interactions [14]. Haisler and colleagues showed that after one day of levitation of human hepatocytes (HepG2 cell line), the MNP appeared to clump together, and cells started to aggregate and grow into 3D shapes around them. In the following days, the spheroids became mature and the changes in the morphology and shape were minimal. Moreover, the authors also demonstrated that the m3D model maintained the epithelial phenotype and function [13].

As expected, the histological evaluation of homotypic spheroids produced only with human non-tumoral cells showed an organized structure and morphology with cells being homogeneously distributed [39]. However, the heterotypic spheroids produced with mice pancreatic tumoral cells and human non-tumoral cells displayed a heterogeneous organization and random distribution of cells and ECM content within the spheroids, with no pattern of regionalization between the different cell types [23,30]. This will promote cell–cell and cell–ECM interactions among the different cell players of the human tissue, mimicking the in vivo physiological and microarchitecture features.

It is well known that cells acquire a different shape and morphology cultured in 2D or 3D. For example, DPSCs displayed a typically flattened shape in 2D culture, whereas in the 3D culture they had a polygonal shape, forming cellular junctions and projections when aggregates were formed [17]. Moreover, a decrease in the cell proliferation index was observed in m3D cultures compared to 2D cultures, which could be associated with exposure to the magnetic field, since it is also able to control the cell culture shape [15], [22]. The differences observed in the cell viability between the cells in the periphery and the center of m3D structures, namely for cultures with more than 2.5 × 10^5^ cells, suggest that the intercellular communications within the 3D structures are most likely different from those observed in conventional 2D cell cultures [19]. Moreover, there are also differences between magnetic-based and magnetic-free 3D cultures, with the magnetic-based 3D structures being more cohesive and compact, with more lipid droplets and extracellular vesicles, a better differentiation performance (after 7 days), and an amelioration of apoptotic effects [17].

Some of the studies reported an early spheroids’ diameter size reduction, which was most probably due to the strong cell–cell and cell–ECM interactions as well as epithelial cell packing, causing cell contraction [17,24,27]. Adine et al. [27] also observed this reduction from 15 min to day 3 of culture, the period corresponding to the spheroid formation stage. Tseng and colleagues [24] observed that after 12 h of spheroid levitation, the planar size of the heterotypic cultures decreased significantly and then remained constant for the next 60 h. The spheroids developed by Chan et al. also experienced a sharp diameter decrease within the first 3 days, but then the diameter remained stable for the rest of the culture period [17].

### 3.9. m3D Cultures to Produce Tumor Spheroids

Among the studies revised, only seven developed tumor spheroids. Two studies developed pancreatic ductal adenocarcinoma (PDAC) m3D spheroids, both to construct a model able to reproduce the microenvironment and cellular interactions of PDAC and to evaluate possible therapies [26,30]. There was also one study devoted to creating ovarian cancer spheroids, with the main goal of analyzing the immune cell interactions and the retrieval of lymphocytes in the tumor microenvironment [15]. Souza and colleagues [22] developed glioblastoma spheroids and the authors observed not only morphological similarities, but also a molecular similarity to orthotopic human tumor xenografts from immunodeficient mice. Reinhardt et al. also produced glioblastoma m3D structures to develop wound dressings and maximize the healing process [33]. Menegakis and his team developed m3D cultures using retinal and skin squamous cell carcinoma lines to study the quiescence induction under hypoxia and its HPV-driven prevention. Vu and colleagues developed an m3D culture of skin squamous cell carcinoma and primary fibroblasts to develop a 3D model of the human skin [32,34]. The majority of the tumor models were obtained using heterotypic cultures since they allow for a better replication of the tumor microenvironment. This microenvironment should include the following characteristics: a necrotic region, a distribution of viable cells with a higher number in the periphery than in the center, a complex cell–cell arrangement, and an active production of the extracellular matrix by the stromal cells [30]. Apart from the general published protocol presenting the common procedures to generate homotypic m3D cultures for lung, kidney, breast, skin, and prostate cancer, a low number of studies using m3D was found, especially presenting the characteristics and the optimization details made to develop the 3D aggregates. The use of the m3D systems to establish heterotypic tumor spheroids is not widely used, however, their optimization may present several benefits for cancer research.

## 4. Limitations

The conduction of this review presented an overview regarding the applications of the m3D system to recapitulate diverse structures of the human system. Despite the increasing application of m3D systems for diverse health research purposes, its application for cancer research is still limited. These m3D systems showed to be useful to recapitulate the normal human physiological properties, with a variety of systems developed. There are general protocols published for different cell lines; however, several modifications to the protocol are often made by the authors in order to optimize the protocol according to their research goal. One of the major concerns pointed out was the difficulty in choosing the most adequate culture medium for the heterotypic m3D structures since it is critical to assure the preservation of all cell phenotypes and their survival [23,24]. According to Leonard et al., when developing 3D cultures of different cell types, the spheroids should be maintained in the cell medium of the most demanding and sensitive cell type, which could be challenging to achieve [36]. Moreover, scarce information was available on the medium used in the experiments by the authors. In addition, there is not specific information regarding the concentration of MNP used in each experiment, with authors not reporting specifically if they used the concentration recommended by the manufacturer or an optimized concentration. The incubation period of cells with the MNP is also missing in the methodology section of some studies included. Sometimes it is not clear if the authors used the magnetic or bioprinting method or when each one was applied during the experiments. The details regarding the production of heterotypic m3D culture would benefit from more details on the several steps of incubation with different cell types, and the cell density of each cell type.

## 5. Conclusions

Cell cultures in 3D structures mimic better the in vivo cellular microenvironment, namely the cell–cell and cell–ECM interactions, and their morphological, physiological, and transcriptional responses. Thus, the 3D cultures emerge as a bridge from conventional 2D cell cultures to in vivo experiments and human clinical trials, allowing for the reduction in animal experiments [1,14]. The 3D cultures also provide a more proper model for cell growth, mimicking in vivo signaling pathways, gene expression, molecular mechanisms, and 3D structure [35].

M3D cell cultures are one of the most recent methodologies used for generating 3D structures. The analysis of selected studies showed that this method constitutes a good cost-benefit approach, which allows for rapid and easy 3D spheroids formation driven mostly by the presence of the magnetic field, with no MNP-induced damage in cell populations. Although the use of MNP and the magnetic fields did not influence the viability of the cells, their use could influence cell morphology and the final shape of the m3D structure archived. The use of m3D systems has been increasing in the last years, most using levitation and bioprinting methods. The magnetic ring structures seem to be an emerging tool for generating bigger 3D aggregates. Although the bioprinting method was the most preferred by the authors of the articles revised, it was also quite common to use a combination of the three methods for the development of 3D structures.

The m3D system could be used either for the development of homotypic or heterotypic 3D cultures, to mimic healthy normal tissue or diseases. In the heterotypic m3D cultures, the authors generally used a combination of cells from the tissue of interest (tumoral or non-tumoral) and stromal cells to promote a better representation of the in vivo microenvironments, inclusive of ECM production. The production of ECM by the m3D structures constitutes an advantage of the model because the use of a scaffold was not needed to obtain spheroids or organoids. However, it was also shown that the m3D models were compatible with several types of scaffolds. Moreover, the capacity of developing long-term cultures with magnetic systems also allows their use instead of animal models. Although the use of m3D for cancer research is still not being widely explored, m3D systems are undoubtedly a tool with great potential for advancement in this important area.

## Figures and Tables

**Table 1:** Search strategy used.

Database	Search Query
Medline (via Pubmed)	Search: (“magnetic nanoparticle *” [Title/Abstract] OR “magnetic levitation *” [Title/Abstract] OR “nanoshuttle *” [Title/Abstract] OR “magnetic bioprint *” [Title/Abstract]) AND (“culture techniques” [MeSH Terms] OR “cells, cultured” [MeSH Terms] OR “Tissues” [MeSH Terms] OR “Organoids” [MeSH Terms] OR “printing, three dimensional” [MeSH Terms]) Filters: Full text, English
Scopus	TITLE-ABS-KEY ((“Magnetic * nanoparticle *” OR “Magnetic levitation *” OR “Nanoshuttle *” OR “Magnetic Bioprint *”) AND (“Cell Culture *” OR “Organ Culture *” OR “Tissue culture *” OR “organoids *” OR “spheroid *” OR “Patient-derived xenografts” OR “Primary Culture *” OR “three-dimensional model *” OR “Three-dimensional cell culture *” OR “levitated culture”)) AND (LIMIT-TO (LANGUAGE, “English”)) AND (LIMIT-TO (DOCTYPE, “ar”))
Web of Science	TS = ((“Magnetic * nanoparticle *” OR “Magnetic levitation *” OR “Nanoshuttle *” OR “Magnetic Bioprint *”) AND (“Cell Culture *” OR “Organ Culture *” OR “Tissue culture *” OR “organoids *” OR “spheroid *” OR “Patient-derived xenografts” OR “Primary Culture *” OR “three-dimensional model *” OR “Three-dimensional cell culture *” OR “levitated culture”)) AND IDIOMA: (English)

## Appendix A

**Table A1:** Summary table of the studies included in the present systematic review and characteristics of each study.

Year	Reference	m3D Model	Heterotypic or Homotypic	Scaffold	3D Mimicking Tissue or Disease	Cell Types	Applications	Observations Regarding m3D Model
2010	[22]	Levitation And Ring Formation	Homotypic and heterotypic	Yes– Magnetic Iron Oxide (MIO)-containing hydrogels	Glioblastoma	(1) C17.2 murine neural stem cells (2) Normal human astrocytes (3) Human glioblastoma (LN-229 or U-251MG) cells	Drug discovery Tissue engineering Biotechnology Regenerative medicine Stem cell research Recapitulation of in vivo protein expression Long-term multicellular studies	- NS concentration: 1 μL/1 cm^2^ of hydrogel (MIO-containing hydrogels) - Petri dish - Period of m3D formation: shorter than 30 min - Final spheroids shape formed at 72–192 h, diameter: 1 mm - m3D cultures had morphological and molecular similarity to orthotopic human tumor xenografts from immunodeficient mice.
2013	[18]	Levitation	Homotypic	Yes–Polylysine-based hydrogel (MagPLL)	Vascular Smooth Muscle Cells System-	(1) Human aortic smooth muscle cells (PH35405A))	In vivo representative system of VSMCs environment Studies related to osteogenic Transdifferentiation of Vascular Smooth Muscle Cells	- Use of MagPLLTM, a polylysine-based hydrogel containing gold and magnetite. MagPLLTM is a previous commercialized version of the MNP, the NanoShuttle™-PL - Magnetite beads were avidly phagocytosed by VSMCs. - m3D levitated cultures proliferated 3–4 times faster with larger calcification clusters than cells in conventional 2D cultures
2013	[19]	Levitation	Homotypic and heterotypic	No	White adipose tissue (WAT)	(1) Mouse 3T3-L1 preadipocyte cells (2) Murine bEND.3 endothelial cells (3) Mouse primary Stromal vascular fraction (SVF).	In vivo representative system of VSMCs environment Adipose Tissue Engineering Physiological interactions between cell populations using functional WAT ex vivo Establishment of adipospheres as a model of WAT development, growth and organogenesis Simulation of vascularization and lipogenesis in cultured tissue Identify molecules bioactive toward individual adipose cell populations WAT transplantation Aid approaches to WAT-based cell therapy	- NS concentration: 8 μL per cm^2^ plate area or ~10,000 cells per mL of MNP, 12 h inbucation - 6 or 24-well flat bottom ULA plates - Magnetic drive maintained during all experiment - Spheroids longevity: up to 45 days - Heterotypic m3D cultures readily formed, with the formation of circular endothelial structures - Adipocytes differentiation is visible with lipids accumulation upon adipogenesis induction.
2013	[13]	Levitation	Homotypic	No	Various-	General protocol for levitation of several cell types: - cell lines, - primary cells - stem cells	Recapitulation of native cellular environments 3D cultures with fine spatial control and in a more complex environment	- Protocol for generate m3D homotypic cultures with applicability also for heterotypic cultures - This protocol details the m3D levitation model and techniques adapted for their analysis. - Optimization of medium volume and cell number depending on cell line.
2013	[38]	Ring Formation	Homotypic	No	Novel 3D assay for drug toxicity screening -	(1) Human embryonic kidney cells (HEK293) (2) Tracheal smooth muscle cells (SMCs)	Drug toxicity screening To predict human in vivo drug toxicity Migration by analysis of m3D ring closure Potential as quantitative assay for high-throughput in vivo toxicity in 3D cultures Potential as a model for wound healing	- NS concentration: 8 μL/cm^2^ cell culture area, overnight incubation - 6 and 96-well ULA plates - −1st levitation of m3D cultures with ECM, and 2nd into 3D ring-shaped cultures - In ring structures, when the magnetic field is removed, the rings close over time allowing the study of migration for 3D cultures
2013	[23]	Levitation	Homotypic and heterotypic	No	Bronchiole-	(1) Human pulmonary microvascular endothelial Cells (PECs) (2) Human epithelial cells (EpiCs) (3) Smooth muscle cells (SMCs) (4) Pulmonary fibroblasts (PFs). All primary human cell	Development of m3D Bronchiole heterotypic cultures (BHC) mimicking the native ECM Magnetic levitation as a tool to create layered and organized cocultures, such as the BCC Investigation of inflammatory responses, angiogenesis and airway remodeling as well as respiratory disorders	- NS concentration: 8 µL/cm^2^ of cell culture surface area or 50 μL/mL medium, overnight incubation. - Homotypic m3D culture: 2 mL of magnetized cell suspensions per Petri dish or 400 mL per well in a 24-well ULA plate (Corning) levitated for 48 h. - Heterotypic m3D cultures: 1st: cell levitation during 4 h; 2nd: spheroids of each cell type were picked up sequentially; 3rd: left on the pen for 4 h; 4th: placed into plates and levitated - The BHC was assembled in hours and exhibited extracellular matrix formation - BHC longevity: 7 days.
2014	[24]	Levitation	Homotypic and heterotypic	No	Aortic valve -	(1) Valvular interstitial cells (VICs) (2) Endothelial cells (VECs) Both from fresh porcine hearts	Development of Aortic valve co-cultures (AVCCs) To understand heart valve biology Applicable in experiments involving mechanobiology or the progression of calcific aortic valve disease Analysis of markers of cellular phenotype, function and ECM. AVCCs possibly have anti-thrombotic potential	- NS concentration: 8 µL/cm^2^ of cell culture surface area or 50 µL/mL medium, overnight incubation - Use of ULA 24-well and 96-well plates for homotypic and heterotypic, respectively - Heterotypic m3D cultures: homotypic spheroids of VICs and VECs were pick up sequentially, and submerge them into 96w ULA plates to forming the AVCCs - Formation of ECM and competent endothelium in AVCCs - AVCCs longevity: 3 days
2016	[36]	Levitation and Bioprinting	Homotypic and heterotypic	No	Breast cancer	(1) cancer cells (2) fibroblasts (3) myofibroblasts (4) immune cells (5) adipocytes From human or animal origin	Protocol to develop 3D model of in vivo breast cancer environment Understanding mechanisms of tumor growth, response to therapeutics and transport of nutrients/drugs Breast cancer research Study of the complexity of tumor microenvironment	- NS concentration: 2–4 µL/cm^2^ - Use of 96w ULA plates - Period of m3D cultures formation: after a couple of hours of incubation
2017	[21]	Levitation	Homotypic	Yes-Poly(urethane acrylate)-poly(glycidyl methacrylate) thermoresponsive nanofabricated substratum (TNFS)	General 3D tissue architecture -	(1) C2C12 mouse myoblasts	Fabrication of tissue-engineered constructs containing complex physiological structures Tissue structure-function relationships Drug screening Disease modeling Regenerative medicine Cellular microenvironments, organization and composition of ECM Creation of structurally ordered tissues	- Low cell density seeding in the TNFS for levitation - High cell seeding in TNFS for cell sheet transfer - NS concentration: 1 μL/10,000 cells in high density, 2 h incubation - NS concentration: 1 μL/10,000 cells in low density, 1 h incibation - Longevity: several days
2018	[26]	Bioprinting	Homotypic	No	Pancreatic ductal adenocarcinoma (PDAC)	(1) DT66066 cells, isolated from KPC-transgenic mice	Simulate complex 3D intercellular interactions New therapeutic approaches	- Use of 24-well flat bottom ULA plates - NS concentration: 8 μL per cm^2^, 12 h incubation - Biocompatibility of the NS validated - NS releasing: 8th day - Longevity: 8 days
2018	[27]	Bioprinting	Homotypic	No	Innervated and bio-functional salivary glands (SG) epithelial cells	(1) Human dental pulp stem cells (hDPSCs)	Formation of bio-functional tissue organoids Epithelial growth Regenerative potential Resembling the function of the SG acinar secretory unit	- NS added at a concentration of 1 µL/2.5 × 10^4^ cells (40 pg/cell). - Use of 96-well ULA plates, 3 × 10^6^ cells/well. - Centrifugation: 1400 rpm, 5 min - Time of formation: 15 min over the magnets, magnets removed after 1–2 h - Longevity: 3 to 11 days - Biocompatibility of the MNP tested
2018	[25]	Levitation	Homotypic and heterotypic	No	White adipose tissue (WAR)–adipospheres -	(1) 3T3-L1 murine embryonic preadipocytes (2) bEND.3 murine endothelial cells (3) Stromal vascular fraction (SVF) cells harvested from primary murine WAT	Protocol for generate WAT-adipospheres Tissue engineering Regenerative medicine Cell-based therapies. Modeling in vivo physiological functions Disease study, such as obesity.	- Use of 24-well cell-repellent plate - NS concentration: 600 µL of NS per T75 flask, overnight incubation - Cell density: 2.4 × 10^5^ cells/spheroid (3T3-L1s); 2.5 × 10^5^ cells/spheroid (95:5 3T3-L1:bEND.3); 3.5 × 10^5^ cells/spheroid (SVFs) - Longevity: 14 days (3T3-L1s homotypic and heterotypic); 21 days (SVFs).
2019	[28]	Bioprinting	Homotypic and Heterotypic	Yes–Hydrogel (fabricated from 10% *w/v* PEGDA, 0.0001% *w/v* TEMPO and a 1.1mM LAP precursor solution using the DLP Pro4500) and Matrigel	Neural microphysiological system-	(1) Spinal cord cells from Long Evans rat embryos, gestation day 15	Microphysiological model of the central nervous system (CNS) Mimic cells of spinal cord and with cell–cell interactions. Preclinical drug screening of neuro-pharmaceuticals	- Use of non-adherent 96-well plate - NS concentration: 1 μL per 60,000 cells, 24 h incubation - Cell density: 0.5 × 10^6^ cells per well - Time of formation: 2 days - Spheroids were placed in scaff - Longevity: 4 weeks (homotypic), 2 weeks (heterotypic)
2019	[30]	Bioprinting	Homotypic and Heterotypic	No	Pancreatic ductal adenocarcinoma (PDAC)	(1) DT66066 pancreatic tumor cells isolated from KPC-transgenic mice (2) Immortalized murine embryonic fibroblasts (iMEF)	Reproducing PDAC for in vitro studies Drug screening and cytotoxicity Study ultrasound therapy (US)-induced cavitation associated with chemotherapy	- Use of 24-well plates with cell repellent surface - NS concentration: 0.15 mg/mL - Cell density: 10^4^ KPC cells per well (homotypic), 10^4^ KPC cells: 2.10^4^ iMEFs per well (heterotypic) - Time of formation: 3 days. - Longevity: 10 days - NS biocompatibility tested: viability decrease 14% (*p* = 0.0014) in fibroblasts with NS
2019	[39]	Levitation	Heterotypic	No	Human Hematopoietic Stem Cells (HSC) Microenvironment -	(1) Human bone marrow-mesenchymal stem cells (MSCs) (2) Umbilical cord blood-hematopoietic stem cells (HSCs) (3) Non-tumoral endothelial cell line	Organotypic multicellular spheres (OMS) mimicking HSC microenvironment Biology of human stem cells Potential in regenerative medicine	- Use of 24-Well ULA Plates - NS concentration: 1 μL per 1 × 10^4^ cells, 12 h incubation - Cell density: 1 × 10^5^ cells/well (MSC: Ec: HSC = 1:5:5 and 1:2:2) - Time of formation: 120 h - Longevity: 15 days - Biocompatibility of the NS tested
2020	[29]	Bioprinting	Homotypic	No	Early embryonic development -	(1) Cow trophectoderm-1 (CT-1) cells	Improve cell attachment and proliferation of the cattle trophectoderm cell line Study early embryonic development.	- Use of flat bottom 96-microwell plates - NS concentration: 38, 56, and 74 μL per well - Cell density: at least 2 × 10^4^ cells per well - Longevity: 7 days - Biocompatibility of the NS tested.
2020	[15]	Bioprinting and Levitation	Homotypic and heterotypic	No	- Immune cell interactions in tumor microenvironment - Low-grade serous ovarian carcinoma	(1) Human ovarian cancer cell line CAISMOV24 (2) Human peripheral blood mononuclear cells (PBMCs)	Model of papillary-like cell aggregates containing lymphocytes. Study of the immune cell interactions	- Use of 24 well ULA plates - NS concentration: 1 µL per 20,000 cells, overnight incubation (CAISMOV24); 1 µL per 20,000 PBMCs in conical tubes, 3 xG centrifugation and resuspension (30 g/5 min) and pipetting cell up and down ~50 times - Cell density: 10^5^ CAISMOV24 cells (homotypic); 1.2 × 10^5^ PBMCs and CAISMOV24 cells (1:5, heterotypic - Time of formation: 3 h - Longevity: 5 days
2020	[31]	Bioprinting	Heterotypic	No	Pancreatic niche -	(1) Human pancreatic β-cell line (EndoC-βH3 cells) (2) VEGF pre-screened umbilical vein endothelial cells (HUVECs) (3) Rat insulinoma INS-1E cells	Study interaction of pancreatic beta (β)-cells with vascular ECs Investigate co-culture effects of human β-cells on human ECs Insulin secretion and the β-cell functionality Improve pre-vascularized transplantable islet grafts	- Use of u-bottom 96-well ULA plates - NS concentration: 40 μL/mL of media, overnight incubation - Cell density: 5000 cells/50 µL - Heterotypic models: (i) “1:1 mix cells”, (ii) “ECs inside” and (iii)“β-cells inside”. - Time of formation: 1 h - Longevity: 5 days.
2021	[17]	Levitation	Homotypic	No	Oral and maxillofacial tissues-mesenchymal stem cells (MSC)	(1) Human dental pulp stem cells (DPSCs) isolated from human teeth	Potential therapeutic efficiency of DPSCs, such as for reconstructing the functions of damaged tissues Study growth and differentiation of DPSCs	- Use of 6 and 24-well ULA plates - NS concentration: 1 μL per 2 × 10^4^ cells, overnight incubation - Cell density: 1 × 10^5^ and 2 × 10^4^ cells/mL - Longevity: 14 days
2021	[14]	Bioprinting	Homotypic	No	Bone tissue-	(1) Human fetal osteoblast cells (hFOB, 1.19)	Bone tissue engineering, such as in the 3D construct in surgery regeneration of mineralized tissue Regeneration of bone defects. Bone formation and regeneration process	- Use of 96-well ULA plates - NS concentration: 100 μL, overnight incubation - Cell density: 5 × 10^4^ cells/well - Time of formation: within hours - Longevity: 14 days
2021	[37]	Levitation and Bioprinting	Homotypic and Heterotypic	No	Tuberculous granulomas–Human Tuberculosis	(1) Human alveolar macrophages (AM) isolated from aspirated bronchoalveolar lavage (BAL) fluid (2) Autologous CD3+ T cells isolated from Peripheral blood mononuclear cells (PBMC)	Model resembling tuberculosis granulomas Study of host/pathogen pathways and immune response involved in the outcome of infection Pharmacological interventions	- Use of 24-well low-adherence plate - NS concentration: 100 µL per 1 × 10^6^ cells - Time of formation: 48 h (innate granuloma) plus 24 h after added CD3^+^ T cells (adaptative granulomas) - At 24 time point is possible to see the assembly of alveolar macrophage - Longevity: 5 days post infection
2021	[32]	Bioprinting	Homotypic	No	Squamous cell carcinoma, osteosarcoma	(1) Human hTert-immortalized retinal pigment epithelium (RPE) cells and derived cell lines (2) Squamous cell carcinoma lines, FaDu, C33A, and U2OS-FUCCI cells (osteosarcoma)	Observation of quiescence induction in hypoxia and its HPV-driven prevention Study of hypoxia-dependent radioprotective phenotype	- NS concentration: manufacture instructions - Cell density: 100,000 cells/mL/well. - The plates were placed overnight in a magnetic frame (Greiner, Bio-One GmbH). - Longevity: 3 weeks - RPE cells failed to grow as 3D spheroids.
2021	[33]	Bioprinting	Homotypic	No	Wound Healing	(1) Human glioblastoma cell line (U87 cells)	Human model of glioblastoma to test wound healing activity of a new hydrogel Development of wound dressings containing the hydroethanolic extract	- Use of cell-repellent 96-well plate - NS concentration: 200 µL per T25 flask, 24 h incubation - Cell density: 100,000 cells/well) - Time of formation: 24 h - Longevity: 5 days.
2021	[35]	Bioprinting	Heterotypic	No	Hepatic inflammatory response -	(1) Chicken hepatocytes (2) Non-parenchymal cells Both freshly isolated from three-week-old Ross-308 male broiler chickens	Establish a proper hepatic inflammatory model To test potential proinflammatory molecules Study hepatic inflammatory homeostasis and stress response	- Use of 96-well cell repellent plates - NS concentration: 500 µL per 5 mL of co-culture suspension (6:1, hepatocyte to non-parenchymal cells), 1 h incubation - Cell density: 100 µL of magnetized co-cultures - Time of formation: 24 h - Longevity: 48 h
2021	[34]	Bioprinting	Homotypic	No	Human skin and their extracellular matrix (ECM).	(1) Primary normal human fibroblasts (NHF) (2) SCC13 cancer cell line (skin squamous cell carcinoma)	To mimic the architecture and ECM of the human skin Analysis of ECM protein regulation, transcriptome, and proteome.	- Use of 6-well cell-repellent plates - NS concentration: 1 µL per 10,000 cells, overnight incubation - Cell density: 1 × 10^6^ cells/well - Longevity: 10 days

Legend: m3D (magnetic 3D cell culture); NS (NanoShuttle^TM^-PL).
